# Understanding Public Judgements on Artificial Intelligence in Healthcare: Dialogue Group Findings From Australia

**DOI:** 10.1111/hex.70185

**Published:** 2025-03-27

**Authors:** Emma K. Frost, Yves Saint James Aquino, Annette Braunack‐Mayer, Stacy M. Carter

**Affiliations:** ^1^ Australian Centre for Health Engagement, Evidence and Values, School of Social Science, Faculty of the Arts, Social Science and Humanities University of Wollongong Gwynneville New South Wales Australia

**Keywords:** artificial intelligence (AI), dialogue groups, focus groups, health care, public views

## Abstract

**Introduction:**

There is a rapidly increasing number of applications of healthcare artificial intelligence (HCAI). Alongside this, a new field of research is investigating public support for HCAI. We conducted a study to identify the conditions on Australians' support for HCAI, with an emphasis on identifying the instances where using AI in healthcare systems was seen as acceptable or unacceptable.

**Methods:**

We conducted eight dialogue groups with 47 Australians, aiming for diversity in age, gender, working status, and experience with information and communication technologies. The moderators encouraged participants to discuss the reasons and conditions for their support for AI in health care.

**Results:**

Most participants were conditionally supportive of HCAI. The participants felt strongly that AI should be developed, implemented and controlled with patient interests in mind. They supported HCAI principally as an informational tool and hoped that it would empower people by enabling greater access to personalised information about their health. They were opposed to HCAI as a decision‐making tool or as a replacement for physician–patient interaction.

**Conclusion:**

Our findings indicate that Australians support HCAI as a tool that enhances rather than replaces human decision‐making in health care. Australians value HCAI as an epistemic tool that can expand access to personalised health information but remain cautious about its use in clinical decision‐making. Developers of HCAI tools should consider Australians' preferences for AI tools that provide epistemic resources, and their aversion to tools which make decisions autonomously, or replace interactions with their physicians.

**Patient or Public Contribution:**

Members of the public were participants in this study. The participants made contributions by sharing their views and judgements.

## 1 Introduction

There is a rapidly increasing number of healthcare applications for artificial intelligence (AI). Recent innovations have been promising for medical image reading, where AI tools have been shown to be effective at tasks such as reading mammograms and screening for lung cancer [1]. Although AI tools have the potential to improve aspects of health care, implementing AI tools into existing healthcare systems also has the potential to impact those systems in undesirable ways. For example, AI systems may not be trained on diverse enough data to detect conditions in marginalised groups, and may therefore exacerbate existing health inequities [2].

There is a growing body of research about public views on healthcare artificial intelligence (HCAI). Our scoping review of this literature found that the majority of research into public views on AI in health care is quantitative [3]. Much of this research has involved either quantifying the extent to which participants are supportive of the introduction of AI [4, 5], or examining whether certain scenarios or features of AI‐enabled healthcare warrant more or less public trust [6, 7]. These studies, including our own survey study [4], often ask participants to indicate their attitude toward AI using semantic or Likert‐style scales measuring public support for [4, 5] or trust in AI [8, 9]. Whilst these studies demonstrate variation in public support for AI in health care, they offer limited insights into why the variation in public support occurs, and how people describe their reasons for their views on HCAI.

Only a small number of qualitative studies have examined public views on HCAI. These studies find that participants are optimistic about the introduction of AI, but with several conditions on their acceptance [10, 11, 12]. People want AI systems to be highly accurate [10], they prefer AI to be in assistive rather than autonomous roles [13, 14, 15], and they do not want their physicians to be replaced by AI [10, 13, 16].

The idea that AI may ‘replace’ physicians is now typically regarded as impractical [17]. Instead, discussions about effective implementation of AI typically centre on how innovations can improve healthcare services by augmenting human intelligence or performing certain healthcare tasks [18, 19]. Even if AI is implemented to assist human physicians, there is still likely to be an impact on people's experiences of healthcare systems and services. With this in mind, we conducted a study to identify the conditions on Australians' support for HCAI, with an emphasis on identifying the instances where using AI in healthcare systems was seen as acceptable or unacceptable. Our research was guided by three questions

1. How do people describe the reasons for their responses to 5‐point semantic scales about their support for AI in health care?
2. Under what conditions do people support the use of AI in health care?
3. Under what conditions do people find it unacceptable to automate a healthcare task with AI?

## 2 Method

Dialogue groups are modified focus groups, designed to support publics to discuss their normative judgements on a research problem [20]. Participants respond to scenarios which encourage them to consider their views [21]. We conducted eight dialogue groups with Australians about the introduction of AI in health care, using a symptom checker scenario as a prompt (Supporting Information: File 1). Ethics approval for this project was approved by the University of Wollongong Human Research Ethics Committee (2022/108).

### 2.1 Participant Recruitment and Selection

We recruited 47 participants across eight groups, via an independent professional recruitment company (Taverner Research), using both social media advertising and random digit dialling. All participants were Australian residents over the age of 18 years.

Potential participants were excluded if they did not have a device with a webcam and internet connection that allowed them to participate via Zoom or if they had worked in health care in the previous 5 years, to avoid knowledge disparities preventing those with less healthcare knowledge from contributing to the groups. Participants who were not capable of participating in the group without the aid of an English language interpreter were also excluded, as we were not able to resource interpreters for multiple languages in a way that would allow natural conversation flow for all participants.

Three separate participant profiles were recruited for the groups (Table 1). Profiles were based on results from a previous analysis conducted by the authors investigating which Australian subgroups were more or less likely to be supportive of the development of HCAI [4]. Profiles were designed to ensure that the project engaged with individuals with a diversity of opinions on HCAI.

**Table 1:** Dialogue group profile information.

Group profile	Description	Rationale^a^	Group #s
A	Aged over 45 50% with chronic health condition or disability (self‐identified)	Older‐than‐millennial age groups were less likely to support the development of HCAI. Those with a chronic health condition/disability were more likely to value human aspects of health care (explainability, knowing who is responsible for my care)	G1
			G4
			G5
B	Aged 45 or under	Younger age groups were found to be more supportive of the introduction of HCAI.	G2
			G3
			G6
C	Has computer science or programming experience	Those with computer science or programming experience were found to be more supportive of the introduction of HCAI. Separated from other groups to avoid those with more technical knowledge dominating discussions.	G7
			G8

Abbreviation: HCAI, healthcare artificial intelligence.

a Rationale for participant profiles adapted from Frost et al. [4].

For Groups 1, 4 and 5, we recruited Australians over the age of 45, ensuring a substantial proportion identified as having a chronic health condition or disability (*N* = 11; 65%). For Groups 2, 3 and 6 we recruited Australians aged 45 or under. For Groups 7 and 8 we recruited 12 Australians with computer science or programming experience. Participants with computer science or programming experience were excluded from participating in Groups 1–6 to prevent knowledge disparities within groups.

Potential participants were contacted by Taverner to ascertain that they met the inclusion criteria and were available to attend one of the groups. EF emailed a copy of the participant information statement to all potential participants, before arranging a phone call to answer any questions and to request participants' verbal consent. After providing consent, participants who were not confident using Zoom were supported to download and test the programme.

### 2.2 Dialogue Group Design

All eight dialogue groups were conducted via Zoom, with EF moderating, and a two‐person co‐moderation team comprised of a research assistant and either YSJA or SMC. Groups took at least 1 h, and up to 2 h. All data were collected in June 2022. Upon joining the Zoom call, participants and researchers participated in a brief icebreaker before the discussion commenced. The discussion was separated into two parts: (1) an initial discussion about AI in general and then about AI in health care and (2) a scenario‐based discussion about a symptom checker tool that uses AI.

In Part 1 of the discussion, Zoom's poll feature was used to present questions to the participants, which were then discussed by the group. Between poll questions, participants were presented with some examples of AI currently in use or in development. These examples were to ensure that participants had a baseline understanding of a range of AI applications, and the extent to which they are used in everyday life (Table 2). We intentionally avoided providing detailed case studies or scenarios in the first part of the groups to encourage discussion about the promises and potential harms of HCAI in general, rather than judgements on individual technologies.

**Table 2:** Group schedule for Part 1 of the dialogue groups.

Prompt	Discussion questions
[Poll question] Pick an option that best describes how familiar you are with 'artificial intelligence' (AI)? 1. Never heard of it 2. Heard of it, but would not be able to describe what it is 3. Know a bit about it 4. Know a lot about it	For those of you who have heard of AI, what have you heard about it? What are some examples that you've heard of that are really exciting? What are some examples that you think are worrying?
Researcher provides some examples of AI used in daily life: Voice assistants like Siri TV and movie recommendations on streaming sites Automatic face tagging on social media sites	
[Poll question] Pick an option to indicate your level of support for the development of AI^a^ 1. Strongly oppose 2. Somewhat oppose 3. Neither support nor oppose 4. Somewhat support 5. Strongly support	Does anyone want to volunteer to explain why they chose the option that they did? Why did you pick [e.g., *somewhat support*] over [e.g., *strongly support*] Now that you have heard other people's reasonings, would anyone like to change their option?
Researcher provides examples of AI used in health care: Interpreting medical imaging results like mammograms Health‐related chatbots Computing someone's risk of lifestyle conditions using their healthcare data	
[Poll question] Pick an option to indicate your level of support for the development of AI in health care 1. Strongly oppose 2. Somewhat oppose 3. Neither support nor oppose 4. Somewhat support 5. Strongly support	Does anyone want to volunteer to explain why they chose the option that they did? Why did you pick [e.g., *somewhat support*] over [e.g., *strongly support*] Did anyone have a response to this question, that was different from your response to the question about your support for AI in general? Any reasons why? Now that you have heard other people's reasonings, would anyone like to change their option?

a Question adapted from Zhang and DaFoe [22] via Isbanner et al. [5] and Frost et al. [4].

In Part 2 of the discussion, we asked participants to reflect on a time where they had concerning and unfamiliar symptoms. Two participants were invited to share their stories if they were comfortable doing so. Participants who shared their stories were asked to reflect on what worked well about the current healthcare system, and what did not work so well. This interlude encouraged participants consider their own personal circumstances and whether they felt that AI would benefit them. Following a brief discussion, participants were shown a video about an AI symptom checker (Supporting Information: File 1).

After the video, participants were asked about their initial thoughts and reactions. Throughout the discussion, the moderator used additional prompts to encourage participants to think about different aspects of utilising a symptom checker app. Example prompts are in Box 1 and the full script is in Supporting Information: File 1; prompts were used to redirect the conversation when required, and therefore not all prompts were used in all groups. Following the groups, participants received an AU$100 gift card to thank them for their time.

Box 1 **Additional discussion prompts.**

Does anyone have any initial thoughts or reactions to this video?

Thinking about the problems or the strengths of the current healthcare system– I'm interested in the ways you think this system might be better or worse than what's in place currently

I'm interested in when you think using an app like this might be appropriate or not appropriate. When do you think it should or shouldn't be used?

We've talked about when it should be used or shouldn't be used—how about you? What kinds of situations do you think you'd be likely to use it for? And are there situations where you wouldn't want to use it?

Let's say you've decided you're going to set this up to use in your house. What sorts of things would you want to know about an app like this before you used it?

A professional transcription company transcribed the audio‐recorded data verbatim. One audio‐recording was lost in a file transfer error. Seven final transcripts and the researcher's comprehensive notes from the lost recording were used in the final analysis.

### 2.3 Analysis

We used Braun and Clarke's [23] reflexive thematic analysis (RTA) to analyse the qualitative data. RTA is a qualitative analysis method which emphasises the researcher's role in the process of theme development [23]. RTA requires the researcher to move iteratively between analysis stages, and remain reflexive about how their methodological and analytical decisions could affect the final results [24].

The first author coded and analysed the data. Analysis followed the process outlined by Braun and Clarke [24]. EF familiarised themselves with the data and took down observations in an analysis journal. Then, EF initially systematically coded the data in Obsidian (https://obsidian.md/).

**Figure 1:**
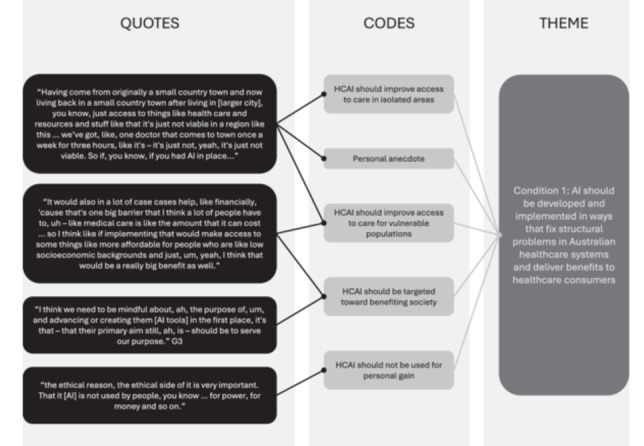
Example of theme development.

Instead of codebook or inter‐rater processes, Braun and Clarke [24] recommend that one researcher codes and themes the data, engaging with co‐authors to reflect on their positionality and assumptions. In regular supervision meetings, EF and SMC discussed EF's positionality, process, interpretive decisions, and theme ideas. Whilst SMC was not involved in the coding process, they co‐moderated several of the groups, and conducted several other dialogue group studies on public views on AI, so were well‐informed enough to challenge EF's decisions and discuss themes. Through these discussions, EF refined the themes and drafted theme structures for discussion with all co‐authors. This process resulted in further refinements to generate the final themes. An example of how quotes were coded and eventually formed into themes is provided in Figure 1. EF analysed the categorical data generated from the polls descriptively, using MS Excel.

## 3 Results

Forty‐seven participants were recruited across eight groups. Participant characteristics are presented in Table 3. Participants were evenly distributed across age and gender categories, nearly half (*N* = 21) were born outside of Australia. Thirteen of the participants (28%) identified as having a chronic health condition or disability. Most of the participants had an undergraduate or postgraduate degree (89%). This limitation is considered further in the discussion.

**Table 3:** Characteristics of recruited participants.

Category	*N*	% (*N* = 47)
**Working status**		
Working full time	19	40%
Working part time	11	23%
Working casually	7	15%
Retired	4	9%
Semi‐retired	1	2%
Unemployed	1	2%
Other (did not specify)	4	9%
**Computer science or programming experience**		
No	35	74%
Yes	12	26%
**Age**		
18–34	16	34%
35–44	9	19%
45–54	7	15%
55–64	6	13%
65+	9	19%
**Gender**		
Female	22	47%
Male	25	53%
**State**		
NSW	25	53%
VIC	9	19%
QLD	6	13%
SA	4	9%
TAS	1	2%
WA	2	4%
**Country of birth**		
Australia	26	55%
Outside Australia	21	45%
**Level of education**		
High School	2	4%
Trade certificate	2	4%
Undergraduate degree	21	45%
Postgraduate degree	21	45%
Unknown	1	2%
**Chronic health condition or disability**		
No	34	72%
Yes	13	28%
		

In our thematic analysis, we identified seven themes to respond to the three research questions. A theme summary can be found in Table 4.

**Table 4:** Thematic analysis results with example quotes.

Research question	Theme	Example quote
RQ1: How do people describe the reasons for their responses to 5‐point semantic scales about their support for artificial intelligence (AI) in health care	Conditional support for AI in health care: depends how it is being used	‘I picked “somewhat support”. Um, I would have honestly probably said strongly support, but I guess it just comes down to, yeah, what is it being used for … and also privacy, um, and if they're going to store what information they have on you in a – in a safe way, then I think it could be used for some really good things.’ G3
	Unconditional support for AI in health care	‘I was one of the “strongly support” people …I think, you know, we're getting to a stage in our – the development of humanity where we need to rely on technologies that are going to surpass us in certain ways.’ G3
RQ2: Under what conditions do people support the use of AI in health care?	AI should be developed and implemented in ways that fix structural problems in Australian healthcare systems and deliver benefits to healthcare consumers	‘It would also in a lot of case cases help, like financially, cause that's one big barrier that I think a lot of people have to, uh – like medical care is like the amount that it can cost … so I think like if implementing that would make access to some things like more affordable for people who are like low socioeconomic backgrounds and just, um, yeah, I think that would be a really big benefit as well.’ G6
	AI should be controlled to prevent harms to patients	‘…then you're going to run into problems because we're a multicultural society. And how do you treat a Sudanese woman or a Muslim girl or a Buddhist boy, or whatever, when you're not building that into the algorithm, you know? And I just think there's a lot of areas yet to be defined and – and analysed correctly before we let it run away from us.’ G4
	AI should increase patient access to information about their health	‘Overall I think it's great if we can develop systems in hospitals that are going to keep people alive. I'd love to see a day where you just go and get a whole body scan and they say, “Right, this is good and this is bad,” you know?’ G4
RQ3: Under what conditions do people find it unacceptable to automate a healthcare task with AI?	AI should not automate decision‐making about a person's health	‘when the AI gives the diagnosis, does it also give a prognosis? So it's – it says, no man, your cancer's so bad it's not even worth treating you; go away and die, you know? I don't want that decision made by a machine.‘ G4
	AI should not replace physician‐patient interaction in health care	‘it's all, um, good to do some process driven stuff, or some – cut out some, you know, mundane work perhaps, but it's still so important to have that human interaction and to speak to a doctor, for instance, in person, because they might be able to pick up something that you may not be telling them, just by body language.’ G2

### 3.1 RQ1: How do People Describe the Reasons for Their Responses to 5‐Point Semantic Scales About Their Support for AI in Health Care?

**Figure 2:**
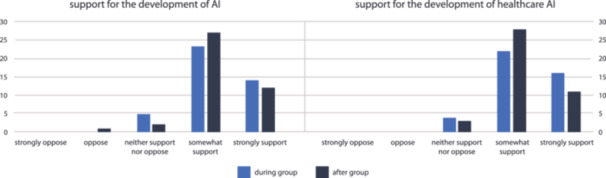
Participant responses to poll questions. AI, artificial intelligence.

During and after the groups, participants were asked three poll questions. Figure 2 shows participant responses, aggregated across the eight groups. Both during and after the groups, most participants reported that they were either ‘somewhat’ or ‘strongly’ supportive of AI and its introduction in health care. However, there was a tendency for participants to move toward the ‘somewhat support’ category by the conclusion of the groups, both from participants initially more supportive and more oppositional to AI.

Participants who were *somewhat supportive* expressed a conditional support for the development and use of AI. It was common for participants to describe AI as something that could be beneficial and useful for themselves and society, so long as certain conditions were met.

‘I picked “somewhat support”. Um, I would have honestly probably said strongly support, but I guess it just comes down to, yeah, what is it being used for … and also privacy, um, and if they're going to store what information they have on you in a – in a safe way, then I think it could be used for some really good things.’

G3 (18–45)

This conditional *somewhat* support was the most common position for participants: only a minority expressed views that more strongly supported or opposed AI. The position of the participants that selected ‘strongly support’ sometimes expressed more certainty that technological development was necessary or inevitable.

‘I was one of the “strongly support” people …I think, you know, we're getting to a stage in our – the development of humanity where we need to rely on technologies that are going to surpass us in certain ways.’

G3 (18–45)

The participants that ‘strongly supported’ the use of HCAI were typically more confident that AI would be developed in ways that would not compromise the patient experience. They were more convinced than other participants that AI would be adequately controlled and would ultimately have a net benefit for society. Strong support was not necessarily unconditional support:

‘I chose “strongly support” too, but I thought the question was, like, very general. There would perhaps be some areas where I would be less comfortable with the development of AI. But then there would be a whole lot of areas where, um, I think that it will continue to expand. Yeah … like, you wouldn't like to see the lines completely blurred between an interaction with a human and an interaction that is AI‐driven.’

G5 (45+)

In contrast, those who were less supportive of AI expressed more concern about its risks and whether enough controls were in place to ensure that HCAI was implemented safely. The following quote is from a participant who selected ‘neither support nor oppose’, who reported feeling both strongly in support of AI's benefits and strongly opposed to its risks.

‘I'm at both ends, I'm strongly opposed to it because of its intrusive nature, and I strongly support it because it might pick up a shadow on my lung or the lump in my brain or a mole on my back. So I'm actually feeling more polarised.’

G1 (45+)

#### 3.1.1 Support for AI in General Versus Support for AI in Healthcare

Participants were asked to discuss their level of support for both AI in general, and AI in health care throughout the groups. We also specifically prompted participants to consider whether they felt more or less supportive of AI in health care, versus AI in general.

Some participants felt more supportive of AI when it was used in health care, because they saw health care as a potential use of AI where the good could outweigh the harms.

‘I said strongly support instead of somewhat support. I think when it comes to healthcare, I just feel like obviously that is one of the most, or if not the most important field, so depending again on how it's going to be used, um, you know, it could make a huge difference in this field.’

G3 (18–45)

Others felt less supportive of the use of AI in health care versus AI in general. These participants saw health care as an area where AI could potentially cause harms if misused. They were often concerned that the use of AI might create information that could be used by insurance companies to discriminate against people with certain health conditions, or that it might make healthcare services too ‘mechanical’ (G4, 45+). Regardless of whether they felt more or less supportive of AI in health care, participants typically saw health care as a high‐stakes field for AI.

### 3.2 RQ2: Under What Conditions do People Support the Use of AI in Health Care?

In the second part of the groups, we asked participants to consider a time when they or a loved one had symptoms and were unsure about the cause or diagnosis. We showed the participants a symptom checker tool as a case study and asked them to consider the circumstances under which they would support this use of HCAI. In our thematic analysis of the transcripts, we identified three main conditions under which the participants supported the use of HCAI.

Condition 1 AI should be developed and implemented in ways that fix structural problems in Australian healthcare systems and deliver benefits to healthcare consumers.

The participants were optimistic about AI's potential to improve healthcare systems, and they wanted AI to be used where it benefited the public. The participants identified problems in Australia's existing healthcare system, such as limited access to healthcare services in remote areas and increasingly expensive care. They speculated that AI could be developed and implemented to solve these problems.

‘Having come from originally a small country town and now living back in a small country town after living in [larger city], you know, just access to things like health care and resources and stuff like that it's just not viable in a region like this … we've got, like, one doctor that comes to town once a week for three hours, like it's – it's just not, yeah, it's just not viable. So if, you know, if you had AI in place…’

G3 (18–45)

‘It would also in a lot of case cases help, like financially, cause that's one big barrier that I think a lot of people have to, uh – like medical care is like the amount that it can cost … so I think like if implementing that would make access to some things like more affordable for people who are like low socioeconomic backgrounds and just, um, yeah, I think that would be a really big benefit as well.’

G6 (18–45)

Despite hoping that AI would be implemented in ways that fixed structural problems and benefited healthcare consumers, the participants were sceptical of whether those developing AI tools had the public's interests at heart.

‘Just say if it was a, like a company, like, I don't know, Amazon or something that owned it, would their recommendations then be like biased towards products that make them money instead of what could be the best outcome for the patient?’

G6 (18–45)

The participants were not necessarily opposed to private companies developing AI tools, but they were opposed to AI tools being developed and implemented because of 'monetary incentives' (G3, 18–45) rather than as a result of identifying areas where the health system would benefit from the integration of AI. Overall, the participants wanted to see AI being implemented where it benefited healthcare consumers or targeted longstanding structural problems in the Australian health system.

Condition 2 AI should be controlled to prevent harms to patients.

Although they were optimistic about AI's potential, the participants felt strongly that AI needed to be controlled to prevent harms to patients and publics. This was the only condition where the participants in the groups with IT or coding experience had different views to participants in the other groups. Participants in the groups with IT experience were quite specific about wanting stricter controls on data storage and sharing, as well as more transparency about the data used to train AI systems and the likely accuracy of outputs. They wanted these controls to be put in place “*at the beginning, not after it's been misused*” (G7, IT experience). These participants did not feel vulnerable themselves, but often framed these regulations as a way to prevent those without IT experience or knowledge from being harmed by HCAI.

‘the more IT literate people are, the more they know that there is an algorithm behind that device and how unreliable that algorithm might be. But for people with lower IT literacy, it might be dangerous, because they might think that whatever comes out of a computer is accurate, you know?’

G7 (IT experience)

The participants without IT experience also often discussed the perceived need for stricter regulations on HCAI, although in more abstract terms. Unlike the groups with IT experience, they often discussed how the development and use of AI made them feel vulnerable.

‘we're letting it run away, we're creating a monster – potentially a monster.’

G4 (45+)

‘the constant bombardment of suggestions and ads and, you know, like …it, sort of, makes me feel like I can't think for myself anymore.’

G3 (18–45)

They discussed wanting “controls” or “parameters” (G4, 45+) to be put in place in healthcare systems to prevent harms from AI. Although the participants felt underqualified to suggest what the controls should be, they often expressed distrust in the existing system to protect them from the potential harms of AI, and wanted more assurance that the right controls were in place to prevent patient harms.

Condition 3 AI should increase patient access to information about their health.

Perhaps the most common reason participants gave for supporting the development of HCAI was that HCAI had the potential to give people access to previously inaccessible information about their health. They felt strongly that AI should enable greater access to information to help patients have control and authority over their health. Participants often described instances where human healthcare workers had failed to make the correct diagnoses.

‘She had been chronically sick for almost two years before she found out from one particular doctor who did one particular test that she was a coeliac. And so, you know, like, it's – it's small things like that like, you know… you can be an expert in your field, but still not be perfect.’

G3 (18–45)

Although the participants admired healthcare workers, they noted that healthcare workers could be biased, and that their expertise could not cover every disease and condition. Participants described instances where they had to receive multiple referrals and visit multiple clinics to consult multiple experts. Many of the participants saw HCAI as having the potential to eliminate errors and extended diagnostic odysseys. They often described this as making diagnoses more ‘objective’ (G3, 18–45) or ‘scientific’ (G1, 45+).

‘medicine is 70% science, 30% art… If this program can, kind of, weed out the art and just give us more scientific, kind of, narrowing down of the symptoms and diagnosis, I think that's really, really clever.’

(G1, 45+)

The participants did not imagine AI replacing physicians, but rather giving patients more information to make decisions about their health. Although the case study was a consumer‐facing tool, the participants typically envisaged AI diagnostic systems being used by GPs or physicians in hospitals to provide access to second opinions and testing that would otherwise require multiple specialist appointments. Participants saw AI being implemented as an additional step or test to ultimately provide patients with information about their health that they would not otherwise have.

‘Overall I think it's great if we can develop systems in hospitals that are going to keep people alive. I'd love to see a day where you just go and get a whole body scan and they say, “Right, this is good and this is bad,” you know?’

G4 (45+)

‘And in important situations when like cancer is concerned, we definitely want to have as many tools in our toolbox to detect this early. And if artificial intelligence shows better results than human judgment, at least like – when you think about like one doctor, like one professional, there might be an error … you don't always have opportunity to have like two doctors or three doctors to have a look.’

G6 (18–45)

Participants were very focused on AI's potential ability to remove some of the uncertainty from medical care by providing more accessible and additional information to patients and their physicians.

### 3.3 RQ3: Under What Conditions do People Find it Unacceptable to Automate a Healthcare Task With AI?

The participants also strongly expressed two conditions under which they felt that it would be unacceptable to automate healthcare tasks.

Condition 4 AI should not automate decision‐making about a patient's health.

Whilst participants were very interested in having access to the informational resources that HCAI could make accessible, they were not comfortable with AI having primary authority. Although the information produced by AI was seen as valuable, even the participants most vocally supportive of AI were uncomfortable with the idea that an AI system could replace the decision‐making capacity of any human healthcare worker.

‘But I think it sounds like everyone's pretty much in agreeance that it's complimentary, it's not a replacement. Like, I mean, the AI checks the human, the human checks the AI at the end of the day.’

G6 (18–45)

The participants spent a large amount of time in the groups discussing the capabilities of AI versus that of humans. They often concluded that healthcare decision‐making required the consideration of more than just data, on which HCAI systems are built. Healthcare workers were seen to be able to consider a patient's best interests and act in ways that the participants saw as admirable.

‘But when they came to the operation, the hospital said, “no, you've got to fit the cheaper part”, which would have caused her more problems than anything, so the doctor made the call not to do the operation. Now, had that been a machine, what would that have done?’

G4 (45+)

AI, in contrast, was seen to be cold and objective, which the participants saw as inappropriate for making sometimes sensitive decisions about diagnoses and treatments.

‘When the AI gives the diagnosis, does it also give a prognosis? So it's – it says, “no man, your cancer's so bad it's not even worth treating you; go away and die”, you know? I don't want that decision made by a machine.’

G4 (45+)

Although HCAI was seen to offer valuable information about healthcare conditions, the participants did not see HCAI tools as systems that should have independent decision‐making capacity in situations where a person's diagnosis or treatment was concerned.

Condition 5 AI should not replace physician‐patient interaction in health care.

In addition to the condition that AI should not make decisions about patients' health, the participants felt that AI should not remove opportunities for physician‐patient interaction. They often justified this position by appealing to human physicians' ability to intuit information or read body language, which they felt AI could not replicate.

‘I remember once I was visiting my friend and her husband's a doctor and he told me interesting things. He said, ah, over time, I learn that so many sentences translates to something, you know, as a doctor. For example, he told me that when someone says, “I feel bad,” usually they have a heart problem… By experience, he realised that it means having a heart problem. But, you know, um, the AI system is dependent on data or rules or whatever it's based on.’

G7 (IT experience)

The participants appealed to a sometimes‐idealised view of empathetic and intuitive physicians to indicate their preference for engaging with other human beings when accessing health care, particularly when they were stressed or had symptoms that they were uncertain about. They emphasised the need for embodiment and human touch when accessing healthcare services, which provided assurance and comfort.

‘last year during COVID lockdown I was incredibly tired so I called my GP and it was just a phone call which you'd think would be very impersonal, but it ended up with me having a big cry to her and I, you know, I don't – that was my first time going to this GP even though it was through a phone … and it was, um, that personal touch, even through a phone, that I don't think artificial intelligence can ever replace.’

G3 (18–45)

‘I can guarantee in the same situation with AI available, I'd be calling that ambulance. I think it's very different when you are talking about a parent, because I don't think anyone's gonna mess around when it comes to their kids. They're going to want someone to touch them, to see them to, you know – you're not gonna trust the computer.’

G6 (18–45)

They emphasised the need for embodiment and human touch when accessing healthcare services, which provided assurance and comfort and made them feel like 'somebody cares' about them (G1, 45+). They felt that this human presence was a crucial element of healthcare services and were very strongly opposed to it being displaced by HCAI.

## 4 Discussion

We conducted eight dialogue groups with Australians about their support for the use of AI in health care. We aimed to identify the reasons participants gave for their responses to 5‐point semantic scales on their support for AI, as well as the conditions under which people found HCAI acceptable or unacceptable.

### 4.1 Participants Were Conditionally Supportive of HCAI, Particularly After Discussing Their Views With Their Peers

By the conclusion of the groups, most participants said that they ‘somewhat support’ the use of AI in health care. In being somewhat supportive of the use of AI in health care, participants recognised that AI could lead to meaningful benefits in health care, but that there were certain use cases that they would not support. Qualitative studies with publics typically have similar findings, that publics are mostly open to the idea of AI being used in health care, but are cautious and uncertain about its potential risks [11, 12]. We found that very few participants in these dialogue groups diverted from this conditionally optimistic view. A minority of participants were less supportive than the majority because they were more cautious about whether HCAI would be implemented with the necessary controls to prevent patient harms and a loss in quality of care. A separate minority were more supportive of AI than the majority because they felt strongly that AI would be implemented in ways that were, on balance, beneficial to patients.

When asked to compare whether they supported the use of HCAI more or less than they supported the use of AI in general, participants were divided. Overall, the participants saw health care as an opportunity to have a significant positive impact, but they also identified that risks associated with bias, over‐automation, and privacy were more significant in health care than in other fields where AI might be used.

In regard to their level of support for HCAI, several participants moved toward the ‘somewhat support’ response category throughout the groups, from initial positions that were more supportive or more oppositional. The change in participants' views throughout the group indicates that, when exposed to information and alternative viewpoints on AI, people's views on HCAI are likely to shift. In these groups, we intentionally gave participants an opportunity to learn about HCAI and discuss their views with their fellow group participants. We expected that many participants may not have had an opportunity to learn about HCAI before participating in our study, and we wanted to ensure as much as possible that participants' views were based on correct and realistic information [25]. Given that the process of participating in the group did shift many participants' views, it may be useful for researchers conducting future studies on public views on HCAI to consider incorporating information and discussion into the research process to develop a common understanding of HCAI amongst participants.

### 4.2 Participants Felt That HCAI Innovations Should Benefit Healthcare Consumers, be Controlled to Prevent Harms, and Enable the Delivery of Patient‐Centred Care

In our thematic analysis, we identified the conditions under which participants typically found HCAI acceptable. First, the participants wanted to see HCAI implemented in ways that delivered benefits to healthcare consumers, particularly through targeting structural problems in Australian healthcare systems. They were concerned about whose interests were reflected in innovation agendas, and they preferred to see HCAI targeted toward benefiting healthcare consumers. Engaging publics through democratic consultation and codesign processes can encourage alignment between innovations and the healthcare issues that people want addressed [26, 27]. Public engagement processes on HCAI can be challenging, as publics may not have previously considered their potential benefits and risks [3]. However, processes which are designed to engage participants in these challenging problems, and which integrate learning and capacity‐building exercises into the research methodology, have generated nuanced recommendations on HCAI related issues (e.g., [28]). Similar processes could be used for involving publics in agenda‐setting to identify areas where HCAI implementation could be beneficial.

Second, we found that participants wanted to know that people were protected from any HCAI‐related harms via regulatory or other control mechanisms. When asked, the participants were uncertain about exactly what these controls should be. However, they often expressed a sense of vulnerability and a need for assurance that they would be protected from harms. During the limited discussion group time, we did not incorporate any information or learning with the participants about the potential harms of HCAI or about potential regulatory solutions. A community jury with Australians on AI in health care, which incorporated more extensive learning about HCAI's benefits and harms, found that participants recommended a series of mechanisms to protect patients from harms [28]. These recommendations included mandatory peer‐assessed evaluations of the safety of HCAI tools, independent bodies to oversee these evaluations, and mandatory transparency reporting of the HCAI tools' efficacy. Incorporating recommendations such as these into regulatory systems may both prevent harms to patients and increase public confidence in Australian regulatory systems.

Thirdly, the participants wanted HCAI to give people access to more epistemic resources about their health. The participants described the process of getting diagnoses and information about their health as drawn‐out and challenging. They saw AI as a potential opportunity to make personalised information more accessible to patients. In suggesting that AI should provide patient access to more epistemic resources, the participants envisaged a healthcare system based on a model of patient‐centred care, where patient autonomy is prioritised by equipping patients with the necessary knowledge about their health to make more informed decisions [29]. While the potential for AI to support patient‐centred care has been discussed in the AI ethics literature [30], these discussions rarely focus on AI's potential to enhance patients' autonomy via enabling access to more epistemic resources. Our results indicate that this access to additional information may be one of people's main conditions for supporting the use of AI in health care.

Although the case study presented to the participants showed a consumer‐facing symptom checker app, the participants typically envisaged AI being more useful in GP clinics or hospitals. They wanted these tools to provide patient access to more information within the clinical setting, but without requiring appointments with multiple different clinical specialists. We speculate that the participants' preference for HCAI to remain within the clinical setting, rather than to be delivered direct‐to‐consumer via apps, is likely a reflection of their preference for patient‐physician interaction and overarching physician authority to remain unaffected by the introduction of AI.

### 4.3 Participants Valued Human Contributions to Healthcare Systems Beyond What HCAI can Provide

The participants identified two main instances where they did not want AI to automate healthcare tasks. Participants did not want their interactions with healthcare workers to be automated by AI, and they did not want any decision‐making about their health to be automated by AI. Similar findings are common in the literature on public views on HCAI [10, 15, 31, 32, 33], where studies have found that publics often express concerns that HCAI systems may not be as effective or as empathetic as human physicians.

Our study findings indicate that participants saw human healthcare workers as valuable for more than just their efficiency and their ability to be empathetic. Participants described instances where they wanted a person physically present to examine a loved one's symptoms, or where a healthcare worker acted in a patient's best interests to make a difficult decision about their health. Their descriptions of why healthcare workers are valuable appealed to very fundamental traits of being human, including the presence and embodiment of a human person, and the capacity to make decisions with practical wisdom [34].

A number of studies have recommended that HCAI tools should be developed to appear more empathetic to more closely resemble care delivered by humans [31, 32, 33]. Our findings, however, suggest that if AI were to emulate human‐like empathy, this would not be sufficient for people to support the replacement of human healthcare workers in their decision‐making capacity or in physician–patient interactions. Instead, the participants felt that HCAI should be implemented as an epistemic tool to give patients and their physicians access to more information about their health.

### 4.4 How Can HCAI be Developed and Implemented in Australia to Meet Public Needs?

A significant effort is currently underway in Australia to develop more effective mechanisms for regulating AI. A whitepaper released in 2023 by the Australian Government Department of Industry, Science and Resources described ‘low levels of public trust’ in AI as a factor impeding the adoption of AI technologies in Australia [35].

Our findings suggest that Australians are not necessarily distrustful of AI. The participants were mostly open to the use of HCAI, but they were often unsure whether enough controls were in place to prevent unintended harms. There is an opportunity for more public engagement to align Australia's AI policy with public interests, which may help people feel more confident in HCAI tools. These engagement approaches may be more effective when they provide participants with opportunities to learn about and discuss AI's benefits and risks, as they may not have had an opportunity to learn about AI before participating.

Overwhelmingly, we found that Australians prefer a healthcare system where important decisions are made by human healthcare workers, and where people have an opportunity to interact with their physicians. It is unlikely that publics will support the use of HCAI tools that displace physicians. Instead, our findings indicate that people hope HCAI tools will increase their access to diagnoses and information about their health without taking away human healthcare workers' decision‐making authority or opportunities for patient‐physician interaction. To implement and use HCAI in ways that publics support, these key conditions should be considered.

### 4.5 Limitations

Our study has some limitations. First, whilst we were successful in recruiting participants diversely across a range of indicators such as age, working status, and country of birth, our final sample lacked sufficient representation from some groups. We excluded those who could not participate without the aid of an English language interpreter. Future research should aim to examine the views of Australian residents who do not speak English, as their concerns about AI may differ to the English‐speaking population. The sample was disproportionately comprised of Australians with a bachelor degree or higher (89%; *N* = 42), compared to the Australian population aged 15–74 (36%) [36]. Given evidence that education levels are often associated with differing views on AI [3], this may limit the generalisability of our findings to the broader population. In addition, our recruitment company did not allow prospective participants to identify with an alternative gender category, which may have inadvertently excluded gender diverse Australians from participating. Future research on HCAI should aim for better representation from underrepresented groups to address the views of gender diverse Australians and Australians without higher education degrees (e.g., [28]).

The symptom checker case study used in the latter part of these groups is one example of many ways that AI could be used in health care. Since this symptom checker tool was a conversational agent marketed to the public, this likely prompted participants to consider the benefits and risks of this type of AI tool. Using multiple case studies may have elicited discussion of different conditions; however we made the decision to focus on one case study to facilitate meaningful and in‐depth discussion.

## 5 Conclusion

Our findings indicate that Australians are conditionally supportive of the use of AI in health care. They support the use of HCAI when it is adequately controlled, implemented to solve existing issues in healthcare systems, and provides patients with greater access to information about their health. The participants did not support the use of HCAI that limits patient‐physician interaction or takes away the decision‐making authority of healthcare professionals. Those in charge of safely implementing HCAI tools should consider involving publics in designing legitimate policy approaches, and implementing HCAI tools in ways that allow publics access to epistemic resources, rather than as replacements for physician judgement or interaction.

## Author Contributions

**Emma K Frost:** conceptualization, methodology, software, data curation, investigation, validation, formal analysis, visualization, writing–original draft. **Yves Saint James Aquino:** conceptualization, supervision, writing–review and editing. **Annette Braunack‐Mayer:** conceptualization, writing–review and editing, supervision. **Stacy M Carter:** funding acquisition, conceptualization, methodology, investigation, supervision, project administration, writing–review and editing.

## Ethics Statement

This study was approved by the University of Wollongong Human Research Ethics Committee (2022/108). Participants provided verbal consent before participating in the groups.

## Conflicts of Interest

The authors declare no conflicts of interest.

## Supporting information

Supporting information.

## Data Availability

The authors have nothing to report.
