# Supplementary material for: Understanding Public Judgements on Artificial Intelligence in Healthcare: Dialogue Group Findings From Australia
Source: Health Expect. 2025 Mar 27;28(2):e70185. doi: 10.1111/hex.70185 (PMC11949843; doi:10.1111/hex.70185)
Supplement: Supplementary file 1 — Supporting information. [file HEX-28-e70185-s001.docx]

**Supplementary File 1 – additional notes on method**

The participants were shown the following video as their symptom checker scenario prompt:

<https://www.facebook.com/watch/?v=1844310612364655>

The video shows a man interacting with a voice assistant symptom checker tool. He explains that he has muffled hearing and an ear ache. The voice assistant asks him follow-up questions about additional symptoms. After the man answers those questions, the symptom checker says that he likely has a middle ear infection.

The following box contains a comprehensive list of probes used to understand participants’ views on the symptom checker scenario.

| - Does anyone have any initial thoughts or reactions to this video? - Thinking about the problems or the strengths of the current healthcare system– I’m interested in the ways you think this system might be better or worse than what’s in place currently - I’m interested in when you think using an app like this might be appropriate or not appropriate. When do you think it should or shouldn’t be used? - We’ve talked about when it should be used or shouldn’t be used – how about you? What kinds of situations do you think you’d be likely to use it for? And are there situations where you wouldn’t want to use it? - Let’s say you’ve decided you’re going to set this up to use in your house. What sorts of things would you want to know about an app like this before you used it?   - Why is that so important?   Let’s say people start to use these symptom checkers instead of doing the kinds of things you described – googling, or calling the GP office, or turning up to hospital. I’d like you now to think about the way things currently are versus people using a symptom checker app.   - I’m especially interested in how accurate the symptom checker app needs to be in giving you the right answer about what condition you might have, or what you should do. What are people’s thoughts about how accurate the app should be?   - what if it was being formally incorporated as a first-line tool in the health system – so it was endorsed by your state government and was recommended as the first thing you check. What would your expectations be then?] - What if the app can tell you what the algorithm recommends, but not *why* it’s making that recommendation?   - OR – how important would it be that people understand why the app is making the recommendation that it is? Why is that? - The app could make mistakes in two directions. For example, it could be risk averse, and tell people to call an ambulance when they don’t really need to, or it could under-estimate risk and tell people they are less seriously ill than they are. What do you think about balancing these two approaches to risk?   Now, I want you to imagine that the app is really popular, and that more and more people are using the app when they have worrying symptoms to tell them what they should do – people are using the app instead of doing all of those things we discussed earlier, like calling the GP or turning up at the hospital. Let’s imagine that the app gets really good at its job – it almost always gives people the right answer about what condition they have or what they should do next.   1. The app relies on technology like Amazon’s Alexa or a computer to run. We know that not everyone has the same access to this kind of technology. If we think about these apps as a future part of the health system, how do you think we should approach this issue of access to the technology?    1. Probe if needed: Some people say that it’s not fair to rely on a healthcare technology that only some people can afford or use. What do you think about those arguments?    2. Probe: Some people say that it doesn’t matter whether everyone can access a technology – as long as it works for those people who can afford or use it. What do you think about those arguments?    3. You could summarise their positions and ask for any further input here |
| --- |
